# Synthesis of Novel Indolyl-Pyrimidine Antiinflammatory, Antioxidant and Antibacterial Agents

**DOI:** 10.4103/0250-474X.41457

**Published:** 2008

**Authors:** S. S. Panda, P. V. R. Chowdary

**Affiliations:** Department of Pharmaceutical Chemistry, Manipal College of Pharmaceutical Sciences, MAHE Manipal-576 104, India

**Keywords:** Indole, pyrimidine, antiinflammatory, antioxidant, antibacterial

## Abstract

A number of chalcones were synthesized by reacting indole-3-aldehyde, prepared by Vilsemeir Haack reaction with 4-substituted acetophenone in NaOH solution in ethanol. These chalcones were immediately reacted with urea, thiourea and guanidine hydrochloride in presence of concentrated hydrochloric acid as reagent to obtain the corresponding hydroxy, thio and amino pyrimidines. The synthesized heterocyclics were characterized on the basis of physical, chemical tests and spectroscopic data and were tested for the acute antiinflammatory activity, antioxidant, antibacterial activity using carragenan-induced rat paw oedema method, DPPH (diphenylpicrylhydrazyl) radical scavenging method and cup plate method using Muller-Hinton agar media respectively. Evaluation of the compounds revealed remarkable antiinflammatory activity reflected by their ability to reduce the carragenan-induced inflammation in rats, appreciable antioxidant activity and also antibacterial activity was observed.

Due to interesting activity of various substituted pyrimidines as biological agents^1^–^3^, considerable attention has focused on this class. The pharmaceutical importance of these compounds lies in the fact that they can be effectively used as analgesics, antiinflammatory, anticonvulsant, insecticidal, herbicidal, antitubercular, anticancer and antidiabetic agents. The ability of indole to exhibit antiinflammatory, antimicrobial, antifungal activities^4^–^6^ prompted the selection of indole as starting compound.

In the light of these interesting biological activities, it appeared of interest to synthesize some new hydroxy/thio/amino pyrimidine derivatives. Indole-3-aldehyde (2) prepared by Vilsemeir Haack reaction from indole (1) was reacted with 4-substituted acetophenones (a-j) in ethanolic NaOH to obtain chalcones (3a-j), which were condensed with urea, thiourea and guanidine hydrochloride in presence of cyclising agent, concentrated hydrochloric acid to obtain hydroxy pyrimidines (4a-j) thio pyrimidines (5a-j) amino pyrimidines (6a-j) respectively. The synthetic sequence leading to the formation of targeted compounds is depicted in scheme 1.

**Scheme 1:**
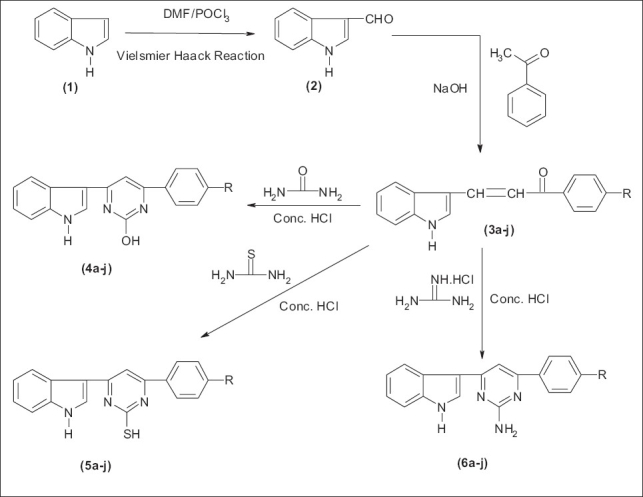
Synthesis of bioactive molecules

The constitution of the products has been supported by element analyses, chemical tests, IR, NMR and Mass spectral data. All the compounds were evaluated for antiinflammatory activity using the carrageenan-induced rat paw edema model, antioxidant activity using DPPH and antibacterial activity against four different strains of *Staphylococcus aureus, Bacillus subtilis, Pseudomonas aeruginosa* and *Escherichia coli* at different concentration of 5 μg/ml, 10 μg/ml, 50 μg/ml and 100 μg/ml.

## MATERIALS AND METHODS

All the melting points were determined using open capillary tubes in scientific melting point apparatus and are uncorrected. UV values were obtained by using Shimadzu UV/Vis Spectrophotometer, UV-1601PC, at Manipal College of Pharmaceutical Sciences, Manipal. IR spectra of the synthesized compounds were scanned using a Shimazdu-Fourier Transform Infrared Spectrophotometer-8300 and KBr press at Manipal College of Pharmaceutical Sciences. PMR spectra of the synthesized compounds were recorded on 300 MHz spectrophotometer at IISC Bangalore. Mass spectra of the synthesized compounds were recorded on Shimazdu-GC-MS QP 5050A at Manipal College of Pharmaceutical Sciences. Progress, purity of the reaction and the intermediates were analyzed using precoated TLC plate and using UV chamber. Male Wistaar rats (150-200 g) were procured and the antiinflammatory activity was carried out on approval of Institutional Animal Ethics Committee, KMC, Manipal.

### Indole-3-aldehyde (2):

In a 100 ml round bottom flask, Vielsmeier Haack complex was prepared using phosphorus oxychloride (1 ml) and N,N-dimethylformamide (3.15 ml) and was stirred using magnetic stirrer at 10-15°. Indole (0.01 mol) was dissolved in minimum quantity of N,N-dimethylformamide and added with stirring at 10-15° in Vielsmeier Haack complex. The reaction mixture was allowed to concentrate at 45° and kept for 30 minutes and then poured into ice cold water (100 ml), a clear red coloured solution was obtained, 10% sodium hydroxide (20 ml) was added to get the precipitate. This was boiled for 1 min and filtered, on cooling crystals were formed. Then these crystals were separated by filtration. Recrystallization was done using aqueous DMF. Melting point of obtained product was found to be 192-194°, R_f_0.52 (petroleum ether:ethyl acetate, 80:20), yield 76%.The formation of the aldehyde was confirmed by the Tollen's test and 2,4-dinitrophenylhydrazine test. The compound was characterized by comparing the malting point and PMR with that reported in literature^7^.

### 1-(4-substituted phenyl)-3-indolyl-2-propen-1-one (3a-j):

Equimolar quantities (0.01 mol) of indole-3-aldehyde and substituted acetophenones were taken in 100 ml conical flask and dissolved in 20 ml of ethanol to this (0.03 mol) of NaOH in minimum quantity of water was added. The mixture was stirred on a magnetic stirrer and the reaction was monitored with TLC. Reaction mixture was diluted with water and acidified with concentrated hydrochloric acid. The precipitated chalcone was filtered and recrystallized from absolute ethanol. The purity of chalcones was tested with thin layer chromatography using solvent system: petroleum ether (60-80°):ethyl acetate [70:30]

### 4-(Indol-3-yl)-6-(4-substituted phenyl)-2-substituted pyrimidines:

Chalcone (0.01 mol) and urea, thiourea or guanidine hydrochloride (0.01 mol) were dissolved in absolute alcohol (20 ml). Few drops of concentrated HCl were added and the reaction mixture was refluxed and the reaction was monitored by TLC. After completion of reaction, it was poured into 250 ml of ice cold water and kept for some time. The crude solid was filtered and subjected to column chromatography. Elution with solvent system ethyl acetate/petroleum ether (60-80°)] gave pure compound as white solid. The entire synthetic route has been shown in scheme 1.

### Characteristics of 4a:

IR (KBr) cm^-1^: 3396 (OH), 3341 (NH), 3048 (Ar-H), 1598 (C=N); PMR (DMSO-d_6_, δ in ppm): 11.2 (s, 1H, OH, D_2_O exchangeable), 10.9 (s, 1H, NH, D_2_O exchangeable), 8.3-7.7(m, 5H, Ar-H), 7.6 (s, 1H, pyrimidine proton), 7.3 (s, 1H, indolyl proton), 7.2-6.9(m, 4H, Ar-H),; m/z: 287 [M]^+·^, 185, 171, 116, 102, 77, 54.

### Characteristics of 4b:

IR (KBr) cm^-1^: 3426 (OH), 3401 (NH_2_), 3381 (NH), 3143 (Ar-H), 1608 (C=N); PMR (DMSO-d_6_, δ in ppm): 11.3 (s, 1H, OH, D_2_O exchangeable), 10.8 (s, 1H, NH, D_2_O exchangeable), 8.4-7.7(m, 4H, Ar-H), 7.5 (s, 1H, pyrimidine proton), 7.3 (s, 1H, indolyl proton), 7.1-6.7(m, 4H, Ar-H), 5.2(s, 2H, NH_2_, D_2_O exchangeable ; m/z: 302 [M]^+·^, 185, 116, 77, 54.

### Characteristics of 4c:

IR (KBr) cm^-1^: 3395 (OH), 3341 (NH), 3108 (Ar-H), 1592 (C=N), 530 (C-Br); PMR (DMSO-d_6_, δ in ppm): 11.2 (s, 1H, OH, D_2_O exchangeable), 10.7 (s, 1H, NH, D_2_O exchangeable), 8.3-7.8(m, 4H, Ar-H), 7.6 (s, 1H, pyrimidine proton), 7.3 (s, 1H, indolyl proton), 7.2-6.9(m, 4H, Ar-H),; m/z: 368 [M+2]^+·^, 366 [M]^+·^, 185, 11, 116, 77.

### Characteristics of 4d:

IR (KBr) cm^-1^: 3396 (OH), 3346 (NH), 3047 (Ar-H), 1590 (C=N), 743 (C-Cl); PMR (DMSO-d_6_, δ in ppm): 11.1 (s, 1H, OH, D_2_O exchangeable), 10.9 (s, 1H, NH, D_2_O exchangeable), 8.4-7.7(m, 4H, Ar-H), 7.5 (s, 1H, pyrimidine proton), 7.3 (s, 1H, indolyl proton), 7.2-6.7(m, 4H, Ar-H); m/z: 324 [M+2]^+·^, 322 [M]^+·^, 85, 171, 137, 116, 77.

### Characteristics of 4e:

IR (KBr) cm^-1^: 3426 (OH), 3381 (NH), 3015 (Ar-H), 1611 (C=N); PMR (DMSO-d_6_, δ in ppm): 11.3 (s, 1H, OH, D_2_O exchangeable), 10.8 (s, 1H, NH, D_2_O exchangeable), 9.5 (s, 1H, OH, D_2_O exchangeable), 9.3 (s, 1H, OH, D_2_O exchangeable), 8.3-7.8(m, 4H, Ar-H), 7.6 (s, 1H, pyrimidine proton), 7.3 (s, 1H, indolyl proton), 7.1-6.8(m, 3H, Ar-H); m/z: 319 [M]^+·^, 185, 171, 134, 116, 77, 26

### Characteristics of 4f:

IR (KBr) cm^-1^: 3394 (OH), 3346 (NH), 3058 (Ar-H), 1599 (C=N); PMR (DMSO-d_6_, δ in ppm): 11.2 (s, 1H, OH, D_2_O exchangeable), 10.7 (s, 1H, NH, D_2_O exchangeable), 8.3-7.7(m, 4H, Ar-H), 7.5 (s, 1H, pyrimidine proton), 7.3 (s, 1H, indolyl proton), 7.1-6.9(m, 4H, Ar-H),; m/z: 305 [M]^+·^, 171, 120, 116, 77, 54, 26.

### Characteristics of 4g:

IR (KBr) cm^-1^: 3376 (OH), 3337 (NH), 3067 (Ar-H), 1613 (C=N); PMR (DMSO-d_6_, δ in ppm): 11.3 (s, 1H, OH, D_2_O exchangeable), 10.9 (s, 1H, NH, D_2_O exchangeable), 8.5-7.7(m, 4H, Ar-H), 7.6 (s, 1H, pyrimidine proton), 7.3 (s, 1H, indolyl proton), 7.2-7.0 (m, 4H, Ar-H), 2.2 (s, 3H, CH_3_); m/z: 301 [M]^+·^, 245, 185, 116, 77, 26

### Characteristics of 4h:

IR (KBr) cm^-1^: 3396 (OH), 3221 (NH), 3053 (Ar-H), 1571 (C=N); PMR (DMSO-d_6_, δ in ppm): 11.2 (s, 1H, OH, D_2_O exchangeable), 10.9 (s, 1H, NH, D_2_O exchangeable), 8.1-7.7 (m, 4H, Ar-H), 7.6 (s, 1H, pyrimidine proton), 7.3 (s, 1H, indolyl proton), 7.2-6.9 (m, 4H, Ar-H), 3.8 (s, 3H, OCH_3_); m/z: 317 [M]^+·^, 279, 185, 171, 132, 116, 54, 26

### Characteristics of 4i:

IR (KBr) cm^-1^: 3396 (O-H), 3341 (NH), 3048 (Ar-H), 1598 (C=N); PMR (DMSO-d_6_, δ in ppm): 11.2 (s, 1H, OH, D_2_O exchangeable), 10.9 (s, 1H, NH, D_2_O exchangeable), 9.8 (s, 1H, OH, D_2_O exchangeable), 8.3-7.7 (m, 4H, Ar-H), 7.5 (s, 1H, pyrimidine proton), 7.3 (s, 1H, indolyl proton), 7.1-6.7 (m, 4H, Ar-H),; m/z: 303 [M]^+·^, 185, 171, 118, 116, 77, 26.

### Characteristics of 4j:

IR (KBr) cm^-1^: 3399 (O-H), 3338 (NH), 3088 (Ar-H), 1606 (C=N), 1501 (NO_2_); PMR (DMSO-d_6_, δ in ppm): 11.3 (s, 1H, OH, D_2_O exchangeable), 10.6 (s, 1H, NH, D_2_O exchangeable), 8.3-7.6(m, 4H, Ar-H), 7.4 (s, 1H, pyrimidine proton), 7.3(s, 1H, indolyl proton), 7.2-6.8(m, 4H, Ar-H),; m/z: 332 [M]^+·^, 242, 185, 171, 147, 116, 77, 26.

### Characteristics of 5a:

IR (KBr) cm^-1^: 3238 (NH), 3088 (Ar-H), 1609 (C=N), 822 (SH); PMR (DMSO-d_6_, δ in ppm): 12.8 (s, 1H, SH, D_2_O exchangeable), 10.8 (s, 1H, NH, D_2_O exchangeable), 8.4 (s, 1H, pyrimidine proton), 8.3-7.6(m, 5H, Ar-H), 7.3(s, 1H, indolyl proton), 7.1-6.8(m, 4H, Ar-H); m/z: 303 [M]^+·^, 201, 102, 116, 78, 54.

### Characteristics of 5b:

IR (KBr) cm^-1^: 3424 (NH_2_), 3235 (NH), 3075 (Ar-H), 1611 (C=N), 824 (SH); PMR (DMSO-d_6_, δ in ppm): 12.9 (s, 1H, SH, D_2_O exchangeable), 10.7 (s, 1H, NH, D_2_O exchangeable), 8.4 (s, 1H, pyrimidine proton), 8.3-7.4(m, 4H, Ar-H), 7.3(s, 1H, indolyl proton), 7.0-6.6(m, 4H, Ar-H), 5.31 (s, 2H, NH_2_, D_2_O exchangeable); m/z: 318 [M]^+·^, 201, 187, 117, 77, 36.

### Characteristics of 5c:

IR (KBr) cm^-1^: 3201 (NH), 3067 (Ar-H), 1568 (C=N), 822 (SH), 528 (C-Br); PMR (DMSO-d_6_, δ in ppm): 12.8 (s, 1H, SH, D_2_O exchangeable), 10.7 (s, 1H, NH, D_2_O exchangeable), 7.9 (s, 1H, pyrimidine proton), 8.1-7.5(m, 4H, Ar-H), 7.4(s, 1H, indolyl proton), 7.2-6.9(m, 4H, Ar-H); m/z: 384 [M+2]^+·^, 382 [M]^+·^, 201, 187, 181, 116,77.

### Characteristics of 5d:

IR (KBr) cm^-1^: 3237 (NH), 3089 (Ar-H), 1610 (C=N), 824 (SH), 752 (C-Cl); PMR (DMSO-d_6_, δ in ppm): 12.7 (s, 1H, SH, D_2_O exchangeable), 10.8 (s, 1H, NH, D_2_O exchangeable), 8.4 (s, 1H, pyrimidine proton), 8.2-7.6(m, 4H, Ar-H), 7.3(s, 1H, indolyl proton), 7.2-6.5(m, 4H, Ar-H); m/z: 340 [M+2]^+·^, 338 [M]^+·^, 201, 137, 116, 77.

### Characteristics of 5e:

IR (KBr) cm^-1^: 3230 (NH), 3088 (Ar-H), 1609 (C=N), 825 (SH); PMR (DMSO-d_6_, δ in ppm): 12.7 (s, 1H, SH, D_2_O exchangeable), 10.7 (s, 1H, NH, D_2_O exchangeable), 9.7 (s, 1H, OH, D_2_O exchangeable), 9.5 (s, 1H, OH, D_2_O exchangeable), 8.5 (s, 1H, pyrimidine proton), 8.3-7.7(m, 4H, Ar-H), 7.3(s, 1H, indolyl proton), 7.1-6.7(m, 4H, Ar-H); m/z: 335 [M]^+·^, 187, 134, 116, 77, 54.

### Characteristics of 5f:

IR (KBr) cm^-1^: 3237 (NH), 3076 (Ar-H), 1608 (C=N), 825 (SH); PMR (DMSO-d_6_, δ in ppm): 12.9 (s, 1H, SH, D_2_O exchangeable), 10.8 (s, 1H, NH, D_2_O exchangeable), 8.4 (s, 1H, pyrimidine proton), 8.2-7.6(m, 4H, Ar-H), 7.2(s, 1H, indolyl proton), 7.2-6.9(m, 4H, Ar-H); m/z: 321 [M]^+·^, 201, 120, 116, 77, 34.

### Characteristics of 5g:

IR (KBr) cm^-1^: 3240 (NH), 3090 (Ar-H), 1611 (C=N), 822 (SH); PMR (DMSO-d_6_, δ in ppm): 12.8 (s, 1H, SH, D_2_O exchangeable), 10.8 (s, 1H, NH, D_2_O exchangeable), 8.5 (s, 1H, pyrimidine proton), 8.3-7.6(m, 4H, Ar-H), 7.3(s, 1H, indolyl proton), 7.0-6.7(m, 4H, Ar-H), 2.3 (s, 3H, CH_3_); m/z: 317 [M]^+·^, 201, 187, 166, 77, 54.

### Characteristics of 5h:

IR (KBr) cm^-1^: 3246 (NH), 3089 (Ar-H), 1608 (C=N), 821 (SH); PMR (DMSO-d_6_, δ in ppm): 12.9 (s, 1H, SH, D_2_O exchangeable), 10.9 (s, 1H, NH, D_2_O exchangeable), 8.4 (s, 1H, pyrimidine proton), 8.1-7.6(m, 4H, Ar-H), 7.3(s, 1H, indolyl proton), 7.2-6.6(m, 4H, Ar-H), 3.9 (s, 3H, OCH_3_); m/z: 333 [M]^+·^, 201, 187, 132, 116, 77, 54.

### Characteristics of 5i:

IR (KBr) cm^-1^: 3238 (NH), 3088 (Ar-H), 1614 (C=N), 825 (SH); PMR (DMSO-d_6_, δ in ppm): 12.8 (s, 1H, SH, D_2_O exchangeable), 10.8 (s, 1H, NH, D_2_O exchangeable), 9.6 (s, 1H, OH, D_2_O exchangeable), 8.3 (s, 1H, pyrimidine proton), 8.3-7.4(m, 4H, Ar-H), 7.3(s, 1H, indolyl proton), 7.2-6.8(m, 4H, Ar-H); m/z: 319 [M]^+·^, 201, 187, 156, 118, 116, 77.

### Characteristics of 5j:

IR (KBr) cm^-1^: 3231 (NH), 3096 (Ar-H), 1618 (C=N), 1535 (NO_2_), 824 (SH); PMR (DMSO-d_6_, δ in ppm): 12.7 (s, 1H, SH, D_2_O exchangeable), 10.7 (s, 1H, NH, D_2_O exchangeable), 8.4 (s, 1H, pyrimidine proton), 8.0-7.5(m, 4H, Ar-H), 7.3(s, 1H, indolyl proton), 7.1-6.6(m, 4H, Ar-H); m/z: 348 [M]^+·^, 201, 147, 116, 77.

### Characteristics of 6a:

IR (KBr) cm^-1^: 3412 (NH_2_), 3201 (NH), 3096 (Ar-H), 1610 (C=N); PMR (DMSO-d_6_, δ in ppm): 10.7 (s, 1H, NH, D_2_O exchangeable), , 8.4-7.9 (m, 5H, Ar-H), 7.8 (s, 1H, pyrimidine proton), 7.6-7.5 (m, 4H, Ar-H), 7.3(s, 1H, indolyl proton), 5.9 (s, 2H, NH_2_, D_2_O exchangeable); m/z: 286 [M]^+·^, 184, 170, 102, 116, 77, 54.

### Characteristics of 6b:

IR (KBr) cm^-1^: 3422 (NH_2_), 3201 (NH), 3093 (Ar-H), 1610 (C=N); PMR (DMSO-d_6_, δ in ppm): 10.9 (s, 1H, NH, D_2_O exchangeable), , 8.5-7.9 (m, 5H, Ar-H), 7.8 (s, 1H, pyrimidine proton), 7.6-7.5 (m, 4H, Ar-H), 7.3(s, 1H, indolyl proton), 5.9 (s, 2H, NH_2_, D_2_O exchangeable); m/z: 301 [M]^+·^, 184, 146, 116, 77, 34.

### Characteristics of 6c:

IR (KBr) cm^-1^: 3404 (NH_2_), 3214 (NH), 3096 (Ar-H), 1614 (C=N), 531 (C-Br); PMR (DMSO-d_6_, δ in ppm): 10.8 (s, 1H, NH, D_2_O exchangeable), , 8.4-7.9 (m, 5H, Ar-H), 7.8 (s, 1H, pyrimidine proton), 7.7-7.4 (m, 4H, Ar-H), 7.3(s, 1H, indolyl proton), 6.1 (s, 2H, NH_2_, D_2_O exchangeable); m/z: 367 [M+2]^+·^, 365 [M]^+·^, 184, 170, 116, 77.

### Characteristics of 6d:

IR (KBr) cm^-1^: 3411 (NH_2_), 3215 (NH), 3089 (Ar-H), 1609 (C=N), 728 (C-Cl); PMR (DMSO-d_6_, δ in ppm): 10.8 (s, 1H, NH, D_2_O exchangeable), , 8.4-7.9 (m, 5H, Ar-H), 7.8 (s, 1H, pyrimidine proton), 7.6-7.5 (m, 4H, Ar-H), 7.3(s, 1H, indolyl proton), 6.3 (s, 2H, NH_2_, D_2_O exchangeable); m/z: 323 [M+2]^+·^, 321 [M]^+·^, 184, 137, 116, 77.

### Characteristics of 6e:

IR (KBr) cm^-1^: 3412 (NH_2_), 3203 (NH), 3099 (Ar-H), 1610 (C=N); PMR (DMSO-d_6_, δ in ppm): 10.7 (s, 1H, NH, D_2_O exchangeable), , 8.5-8.0 (m, 4H, Ar-H), 7.8 (s, 1H, pyrimidine proton), 7.6-7.5 (m, 3H, Ar-H), 7.2(s, 1H, indolyl proton), 6.5 (s, 2H, NH_2_, D_2_O exchangeable); m/z: 318 [M]^+·^, 170, 134, 116, 77, 54.

### Characteristics of 6f:

IR (KBr) cm^-1^: 3412 (NH_2_), 3200 (NH), 3095 (Ar-H), 1611 (C=N); PMR (DMSO-d_6_, δ in ppm): 10.9 (s, 1H, NH, D_2_O exchangeable), , 8.3-7.9 (m, 4H, Ar-H), 7.7 (s, 1H, pyrimidine proton), 7.6-7.5 (m, 4H, Ar-H), 7.3(s, 1H, indolyl proton), 6.0 (s, 2H, NH_2_, D_2_O exchangeable); m/z: 304 [M]^+·^, 256, 184, 120, 116, 77.

### Characteristics of 6g:

IR (KBr) cm^-1^: 3416 (NH_2_), 3201 (NH), 3100 (Ar-H), 1610 (C=N); PMR (DMSO-d_6_, δ in ppm): 10.7 (s, 1H, NH, D_2_O exchangeable), , 8.4-7.9 (m, 4H, Ar-H), 7.8 (s, 1H, pyrimidine proton), 7.6-7.4 (m, 4H, Ar-H), 7.3(s, 1H, indolyl proton), 6.9 (s, 2H, NH_2_, D_2_O exchangeable); m/z: 300 [M]^+·^, 256, 184, 170, 116, 77, 34.

### Characteristics of 6h:

IR (KBr) cm^-1^: 3408 (NH_2_), 3220 (NH), 3055 (Ar-H), 1571 (C=N); PMR (DMSO-d_6_, δ in ppm): 10.8 (s, 1H, NH, D_2_O exchangeable), , 8.2-7.8 (m, 4H, Ar-H), 7.7 (s, 1H, pyrimidine proton), 7.3-7.0 (m, 4H, Ar-H), 7.4 (s, 1H, indolyl proton), 6.8 (s, 2H, NH_2_, D_2_O exchangeable); m/z: 316 [M]^+·^, 184, 132, 116, 77, 54.

### Characteristics of 6i:

IR (KBr) cm^-1^: 3422 (NH_2_), 3201 (NH), 3095 (Ar-H), 1615 (C=N); PMR (DMSO-d_6_, δ in ppm): 10.7 (s, 1H, NH, D_2_O exchangeable), , 8.5-7.9 (m, 4H, Ar-H), 7.8 (s, 1H, pyrimidine proton), 7.7-7.5 (m, 4H, Ar-H), 7.2(s, 1H, indolyl proton), 6.1 (s, 2H, NH_2_, D_2_O exchangeable); m/z: 302 [M]^+·^, 184, 170, 118, 116, 77, 54.

### Characteristics of 6j:

IR (KBr) cm^-1^: 3410 (NH_2_), 3216 (NH), 3091 (Ar-H), 1617 (C=N), 1533 (NO_2_); PMR (DMSO-d_6_, δ in ppm): 11.0 (s, 1H, NH, D_2_O exchangeable), , 8.4-7.9 (m, 4H, Ar-H), 7.8 (s, 1H, pyrimidine proton), 7.7-7.4 (m, 4H, Ar-H), 7.3(s, 1H, indolyl proton), 5.9 (s, 2H, NH_2_, D_2_O exchangeable); m/z: 331 [M]^+·^, 184, 170, 147, 116, 77, 54, 35.

### Antiinflammatory activity:

Carrageenan-induced rat paw oedema method was employed for evaluating the antiinflammatory activity of 4f, 4j, 5b, 5h, 6c and 6d at 200mg/kg body weight in Wistar rats (weighing 150-200 g). The rat paw oedema was produced by the method of Winter *et al*^8^. The percentage inhibition of inflammation was calculated by applying Newbould formula^9^. The results were found to be statistically significant against control at P< 0.05 by applying Scheffe's Post Hoc method.

### Antioxidant activity:

All of the thirty compounds synthesized were analyzed for the antioxidant activity using DPPH radical scavenging method.

### Antimicrobial activity:

The compounds 4a-j, 5a-j and 6a-j were screened for their antibacterial activity against pathogenic organisms *Staphylococcus aureus, Bacillus subtilis Pseudomonas aeruginosa* and *Escherichia coli* at concentration of 5 μg/ml, 10 μg/ml, 50 μg/ml and 100 μg/ml using ciprofloxacin as standard. DMSO was used as solvent control, Muller-Hinton agar medium was used as culture medium. The method employed was cup-plate method^10^,^11^. The zones of inhibition formed were measured in mm.

## RESULTS AND DISCUSSION

All the physical characteristics of the synthesized copound are presented in Table 1. The purified products were screened for antiinflammatory, antioxidant and antibacterial activity. Six compounds were screened for antiinflammatory activity using carrageenan-induced rat paw oedema method. Out of them 4f, 5band 6c have shown better antiinflammatory activity than the standard drug, ibuprofen (Table 2). All products were screened for antioxidant activity using DPPH radical scavenging method. Compounds 4b, 4f, 4h, 5b, 5h, 6e and 6h have exhibited good antioxidant activity which is comparable with that of standard drug, ascorbic acid (Table 3). All products were screened for antibacterial activity towards different strains of *Staphylococcus aureus, Bacillus subtilis Pseudomonas aeruginosa* and *Escherichia coli* at different concentration of 5 μg/ml, 10 μg/ml, 50 μg/ml and 100 μg/ml compared to standard drug, ciprofloxacin. The compounds 4b, 4c, 4d, 4f, 4h 4j, 5b, 5c, 5d, 5f, 5h, 5j, 6d, 6f, 6h and 6j showed very good zone of inhibition at the concentration of 5-10 μg/ml against different bacteria (Table 4). Based on the structure activity relationships, it can be concluded that the presence of halogen group at 4 positions i.e. 4c, 4d, 4f, 5c, 5d, 5f, 6d and 6f showed good activity towards gram negative bacteria. Presence of nitro group and methoxy group at 4 position i.e. 4h, 4j, 5h, 5j, 6h, and 6j displayed good activity towards Gram positive bacteria.

**TABLE 1:** PHYSICAL DATA OF SYNTHESIZED COMPOUNDS

Comp. code	R	λ_max_	Yield (%)^a^	M.P (°)	Rf. Value^b^
4a	-H	278	77	146-148	0.58
4b	-NH_2_(p)	261.9	56	198-199	0.60
4c	-Br (p)	252.6	48	201-203	0.62
4d	-Cl (p)	255.6	64	223-225	0.63
4e	-OH (o,p)	279.8	54	226-228	0.64
4f	-F (p)	276.4	46	215-217	0.60
4g	-CH_3_(p)	275.4	35	210-212	0.65
4h	-OCH_3_(p)	277.8	69	286-288	0.59
4i	-OH (p)	280.1	53	185-186	0.56
4j	-NO_2_(p)	281.4	70	256-258	0.61
5a	-H	276.8	72	270-272	0.65
5b	-NH_2_(p)	262.3	65	223-225	0.58
5c	-Br (p)	252.8	65	216-218	0.59
5d	-Cl (p)	255.6	62	239-240	0.61
5e	-OH (o,p)	278.5	76	205-207	0.65
5f	-F (p)	275.6	53	310-312	0.59
5g	-CH_3_(p)	276.6	29	218-220	0.63
5h	-OCH_3_(p)	276.4	68	290-292	0.57
5I	-OH (p)	277.8	46	245-247	0.64
5j	-NO_2_(p)	280.8	76	269-271	0.61
6a	-H	277.8	45	248249	0.66
6b	-NH_2_(p)	264.8	63	225-227	0.65
6c	-Br (p)	255.0	65	256-258	0.63
6d	-Cl (p)	250.8	75	241-242	0.59
6e	-OH (o,p)	279.8	50	295-297	0.58
6f	-F (p)	276.2	48	247-249	0.66
6g	-CH_3_(p)	277.1	25	290-292	0.62
6h	-OCH_3_(p)	277.4	62	223-225	0.58
6i	-OH (p)	276.3	49	201-202	0.56
6j	-NO2 (p)	279.4	62	270-272	0.57

a. Pure isolated compounds

b. petroleum ether:ethyl acetate (75:25)

**TABLE 2:** RESULTS OF ANTIINFLAMMATORY STUDIES OF COMPOUNDS

Group	Dose in μg	Mean edema±SE	% Reduction in edema volume
Control	-	0.406±0.047	-
Ibuprofen	200	0.170±0.019^a^	68.08
4f	200	0.153±0.021^ab^	69.5
4j	200	0.268±0.022^a^	32.9
5b	200	0.105±0.019^ab^	73.7
5h	200	0.188±0.014^a^	52.6
6c	200	0.126±0.014^ab^	70.7
6d	200	0.175±0.026^a^	36.6

Allowance value was calculated by using One-way ANOVA and Post-Hoc (Scheffe) method. Any two means showing allowance value less than 0.05 are statistically significant with respect to control (a) and ibuprofen (b).

**TABLE 3:** RESULTS OF ANTIOXIDANT STUDIES OF COMPOUNDS

Compd.	% inhibition at various concentrations in μg/ml						
	
	1000	500	250	100	50	25	10
4a	62.0	54.3	46.7	39.6	27.6	19.0	13.2
4b	73.1	64.5	52.2	41.3	32.3	26.7	10.2
4c	56.8	49.3	38.8	29.4	20.1	14.6	08.5
4d	49.4	40.2	32.1	25.7	19.7	12.4	06.3
4e	52.5	46.8	37.3	28.9	21.2	16.7	10.7
4f	68.9	57.8	43.2	37.6	29.7	15.1	09.2
4g	50.1	44.8	38.9	33.4	24.3	17.2	09.3
4h	79.6	67.5	60.9	53.5	34.6	24.3	17.6
4i	63.2	56.7	49.8	40.7	29.5	18.3	11.3
4j	50.6	44.8	39.9	30.3	22.8	17.5	10.3
5a	64.3	57.2	47.8	36.5	29.5	19.3	13.2
5b	80.6	67.5	61.9	53.5	34.6	26.3	17.8
5c	55.6	44.5	37.3	25.7	18.6	14.2	10.6
5d	48.9	37.8	29.5	20.7	13.6	10.8	05.5
5e	43.2	33.5	27.4	21.1	17.3	11.4	06.4
5f	58.3	45.9	36.8	28.9	20.7	16.8	11.1
5g	62.3	56.7	45.3	36.7	24.8	16.3	11.3
5h	73.7	66.8	54.5	43.3	30.2	27.8	19.7
5I	54.1	46.3	39.4	27.9	19.8	13.7	08.4
5j	51.5	46.8	35.3	27.9	20.2	17.7	11.7
6a	39.8	33.7	26.8	20.1	14.3	09.8	05.3
6b	54.3	42.5	36.7	25.2	16.6	12.2	09.6
6c	62.8	55.4	42.6	37.7	29.8	21.3	12.1
6d	51.7	46.9	36.8	29.9	21.1	16.9	10.2
6e	79.8	47.9	38.7	28.9	22.3	16.8	11.7
6f	50.7	41.3	32.8	23.2	18.7	11.4	07.3
6g	53.5	46.6	34.3	26.9	21.2	16.8	11.7
6h	78.9	46.8	39.8	27.7	19.6	11.8	07.5
6i	41.2	34.5	27.6	20.5	15.3	10.2	05.1
6j	55.9	43.7	36.3	28.5	21.1	14.3	06.3

**TABLE 4:** RESULTS OF ANTIBACTERIAL STUDIES OF COMPOUNDS ZONE OF GROWTH INHIBITION

Compd.	*S. aureus*		*B. subtilis*		*E. coli*		*P. aeruginisa*	
				
	50*	100*	50*	100*	50*	100*	50*	100*
4a	12	16	-	-	14	16	11	13
4b	22	26	-	09	14	17	17	24
4c	14	20	-	-	12	14	14	19
4d	13	19	-	-	-	10	13	18
4e	-	14	-	-	-	-	10	14
4f	14	18	-	-	14	16	14	17
4g	-	10	-	-	10	15	12	13
4h	14	21	-	-	12	14	13	17
4i	12	16	-	10	-	-	10	11
4j	20	22	13	24	13	16	16	23
5a	10	13	-	-	10	11	10	12
5b	16	19	12	16	11	13	13	18
5c	13	16	-	10	13	18	13	15
5d	10	17	-	-	11	16	10	16
5e	-	-	-	08	-	-	-	09
5f	11	19	-	-	14	17	11	14
5g	12	21	-	-	10	11	-	10
5h	17	25	10	12	15	16	11	13
5I	13	19	11	13	13	15	-	-
5j	10	16	12	19	12	13	15	19
6a	-	10	-	-	11	14	10	11
6b	18	24	14	18	10	12	-	10
6c	17	23	-	-	13	17	13	17
6d	12	19	-	-	10	12	12	15
6e	11	16	-	10	10	13	10	13
6f	16	22	09	13	15	16	17	23
6g	19	14	-	-	-	10	10	13
6h	23	29	12	16	13	15	16	25
6i	18	21	-	-	-	11	11	13
6j	27	31	11	23	12	16	17	24
Ciprofloxacin	29	33	18	21	17	20	18	26

* Indicates concentration of drug in μg/ml. Zone of inhibition in mm
